# Realistic Gene Transfer to Gene Duplication Ratios Identify Different Roots in the Bacterial Phylogeny Using a Tree Reconciliation Method

**DOI:** 10.3390/life12070995

**Published:** 2022-07-04

**Authors:** Nico Bremer, Michael Knopp, William F. Martin, Fernando D. K. Tria

**Affiliations:** Institute for Molecular Evolution, Heinrich Heine University Düsseldorf, 40225 Düsseldorf, Germany; michael.knopp@hhu.de (M.K.); bill@hhu.de (W.F.M.); tria@hhu.de (F.D.K.T.)

**Keywords:** tree reconciliation, species tree rooting, gene transfer rate, gene duplication rate, genome evolution

## Abstract

The rooting of phylogenetic trees permits important inferences about ancestral states and the polarity of evolutionary events. Recently, methods that reconcile discordance between gene-trees and species-trees—tree reconciliation methods—are becoming increasingly popular for rooting species trees. Rooting via reconciliation requires values for a particular parameter, the gene transfer to gene duplication ratio (T:D), which in current practice is estimated on the fly from discordances observed in the trees. To date, the accuracy of T:D estimates obtained by reconciliation analyses has not been compared to T:D estimates obtained by independent means, hence the effect of T:D upon inferences of species tree roots is altogether unexplored. Here we investigated the issue in detail by performing tree reconciliations of more than 10,000 gene trees under a variety of T:D ratios for two phylogenetic cases: a bacterial (prokaryotic) tree with 265 species and a fungal-metazoan (eukaryotic) tree with 31 species. We show that the T:D ratios automatically estimated by a current tree reconciliation method, ALE, generate virtually identical T:D ratios across bacterial genes and fungal-metazoan genes. The T:D ratios estimated by ALE differ 10- to 100-fold from robust, ALE-independent estimates from real data. More important is our finding that the root inferences using ALE in both datasets are strongly dependent upon T:D. Using more realistic T:D ratios, the number of roots inferred by ALE consistently increases and, in some cases, clearly incorrect roots are inferred. Furthermore, our analyses reveal that gene duplications have a far greater impact on ALE’s preferences for phylogenetic root placement than gene transfers or gene losses do. Overall, we show that obtaining reliable species tree roots with ALE is only possible when gene duplications are abundant in the data and the number of falsely inferred gene duplications is low. Finding a sufficient sample of true gene duplications for rooting species trees critically depends on the T:D ratios used in the analyses. T:D ratios, while being important parameters of genome evolution in their own right, affect the root inferences with tree reconciliations to an unanticipated degree.

## 1. Introduction

Species trees are crucial to understanding how biological lineages, their genomes and their traits evolve over time and are central to modern evolutionary research. There are currently several approaches for species tree reconstruction, with most approaches yielding unrooted trees in which the polarity of processes is not resolved. A rooting step (root inference) is hence often required for evolutionary interpretations of species trees. Methods commonly used for rooting include: (i) the outgroup [1], including the special case of tree rooting with ancient gene duplications [2]; (ii) the midpoint criterion [3]; (iii) the minimal ancestor deviation (MAD) approach [4]; (iv) molecular clock models [5]; and (v) time-irreversible models [6]. The accuracy and assumptions underlying these different rooting methods, which can also be used for rooting gene trees, have been investigated and compared to some extent [4,7,8,9,10].

A different approach for rooting species trees has emerged from gene-tree-species-tree reconciliation models [11,12,13,14], referred to in the following as tree reconciliation models for simplicity. These models were originally designed to infer and quantify different evolutionary processes at the level of genes within genomes such as gene duplication, gene transfer and gene loss. The analysis requires a sample of gene trees, a species tree (reference tree), and operates by the measure of topological discordance between individual gene trees and the reference species tree. Gene-tree-species-tree discordances are assumed to arise due to evolutionary processes which are brought into agreement (‘reconciled’) by invoking gene transfer, gene duplication and gene losses as required. The gene trees in the sample may contain paralogues, resulting from gene duplications, and the gene trees do not need to contain all the species in the species tree. In current practice, tree reconciliation models implemented in a maximum-likelihood framework treat each gene tree independently [15] and assign a likelihood score to each gene tree which (i) corresponds to the likelihood of the most likely reconciliation scenario, and (ii) depends upon the root of the species tree: the varying position of the root within the species tree, but not the gene tree topology, generates a different likelihood value for the tree reconciliation process. When a sample of gene trees is used, the distributions of likelihoods as a function of the root in the species tree can be used for root inference. The position of the root is inferred by identifying the root branch that maximizes the likelihoods across all gene trees, which can be assessed, for example, with the approximately unbiased (AU) test [16]. The AU test posits as a null hypothesis that the distributions of likelihoods for the different roots are statistically equal and returns one *p*-value for each tested root. Roots with *p*-values below a predefined threshold (*p*-value ≤ 0.05, for instance) are rejected. While rejection by the AU test means that the given root is unlikely to be correct, many roots may pass the AU test, giving rise to a set of inferred roots at a specified *p*-value threshold.

One important aspect of tree reconciliation analyses is that a ratio of gene transfer to gene duplication rates (T:D) is necessary to distinguish among alternative reconciliation scenarios. The T:D ratio constrains the relative numbers of gene duplications and gene transfers that are inferred when reconciling a gene tree against the species tree. The T:D ratio can be either preset, specified by the user beforehand, or estimated from the data. In practice, T:D is often treated as a variable of unknown value in tree reconciliation analyses, thus automatic estimations are commonplace. However, T:D values automatically obtained from tree reconciliation analyses typically differ by several orders of magnitude from independent estimates, as we will show in this paper. The accuracy of the T:D estimates as well as the impact of T:D on species tree rooting obtained with tree reconciliations are under-investigated issues.

As one example, starting from a species tree of major bacterial groups, Coleman et al. [17] used a popular tree reconciliation program—ALE [14]—to examine support for 62 possible root positions within a bacterial reference (species) tree. They reconciled a total of 11,272 gene trees reconstructed from a dataset with 265 bacterial genomes and reported three likely root positions within the bacterial tree. T:D ratios were estimated individually for each gene tree by ALE, starting from an assumed T:D ratio of 1:1 that serves as a seed to initiate calculations of gene transfer and gene duplication rates during reconciliation. Thus, ALE initially assumes that the rates of gene transfer and gene duplication are equal, but these rates are optimized by the likelihood criterion and the final estimates after reconciliation may differ from the initial 1:1 T:D ratio. For simplicity, we will refer to analyses involving this optimization procedure as 1:1 T:D throughout the text, which is justified by observations that the average optimized T:D values across gene trees are typically close to the initial 1:1 ratio used as seed (see results and discussions). With a prior T:D ratio of 1:1, the AU tests of Coleman et al. [17] rejected 59 out of the 62 root positions considered, and the remaining three formed a confidence root set of neighboring root positions within the reference tree residing between two bacterial clades that they designated as Gracilicutes and Terrabacteria. The only difference between the three roots found by Coleman et al. [17] concerns the relative positioning of the lineage they designated as Fusobacteria. These three roots are the main result of the paper upon which their further inferences about the nature of the last bacterial common ancestor rest. Their findings differed substantially from those of an earlier report about the position of the root in the bacterial tree and the biology of the last bacterial common ancestor [18].

The T:D ratio that ALE estimated for each gene tree is likely to affect the bacterial roots that passed the AU test, but the impact of T:D estimates upon the root results was not reported by Coleman et al. [17]. It is known that gene transfers in prokaryotes vastly outnumber gene duplications and play the dominant role in the growth of bacterial biochemical networks [19,20]. In quantitative terms, and as estimated by reconciliation-independent methods, the frequency of gene transfer in prokaryotes is about 50–100 times higher than the frequency of gene duplications [21,22], whereby the increase in genome size generated by transfers is compensated by the deletion bias inherent to prokaryote genome evolution [23,24,25,26,27,28,29]. In the analysis of Coleman et al. [17] the final T:D ratio differed across gene trees not by a factor of 20, but by 20 orders of magnitude. That is, ALE estimated that gene transfers are roughly 10^10^ times more likely than gene duplications for some genes, whereas gene duplications are 10^10^ more likely than gene transfers for other genes. Such a large variation in T:D ratios across bacterial genes, from 10^−10^ to 10^10^, represents an unrealistically broad range in comparison to previous studies of T:D ratios in real data using methods that are independent of tree reconciliation analyses [21,22].

Here we asked: Does the freedom in T:D ratios affect ALE’s choice of preferred roots? To answer this question, we reanalyzed the bacterial trees from Coleman et al. [17] using ALE under a set of predefined T:D ratios based on analyses of real genome data and repeated the AU tests. Additionally, we analyzed an independent dataset composed of metazoan-fungi species for which the root position is known as a control for our analyses. Although the accuracy (or lack thereof) of the nominal evolutionary rates T and D are likely to affect root inferences made by ALE in their own right, we investigated here the impact of T:D ratios, which are a measure for the relative frequency of gene transfers to gene duplications. We focused on T:D in particular because reliable reference values of T:D ratios could be readily obtained from the literature, whereas reliable values for the nominal rates (T and D) are more challenging to find. We used the ALE-independent T:D estimates as references to assess ALE’s performance on the test datasets.

## 2. Material and Methods

For the bacterial dataset gene trees reconstructed from protein alignments, species trees and the fraction of missing genes per species were kindly provided by T. Williams [17]. The gene trees were reconciled against the rooted species tree using the program ALEml_undated [14] with the same parameters as described in Coleman et al. [17] (1:1 T:D ratio). Note that ALE analyzes each gene tree independently, hence the gene duplication and the gene transfer rates are optimized for each gene tree independently from the others. Additional tree reconciliations with ALEml_undated were conducted as to conform to different gene transfer to gene duplication (T:D) ratios by specifying, for each gene tree, the gene duplication and the gene transfer rates while maintaining the remaining parameters unchanged. Note that while ALE allows users to separately adjust T and D for each gene tree, there is not a single T:D parameter that can be given as input. Hence to obtain a desired T:D value, we proceeded as follows: for a given gene tree, the gene transfer rate was set to the value returned by the 1:1 T:D analysis (for that same gene tree), in which the gene transfer rate was automatically optimized by ALE. To obtain a desired T:D ratio, the gene duplication rate was defined as the division of the gene transfer rate by an adjusting factor as to obtain the desired T:D ratio. For instance, if the optimized gene transfer rate for a gene tree was 0.5 and we wanted to constrain the T:D to 50:1, then we set T to 0.5 and D to 0.01. The range of T:D ratios evaluated for the bacterial dataset were 50:1, 100:1 and 50:1 or more. For the analysis of 50:1 or more, the gene duplication rates were only adjusted for gene trees with an optimized T:D ratio below 50:1.

For the fungi-metazoan dataset, gene families were obtained from EggNOG version 4.5 [23], and it consists of 15,614 gene families spanning 31 species. Protein sequence alignments were generated for each gene family using MAFFT version 7.027b with the L-INS-i alignment strategy [24] Gene trees were reconstructed with IQ-TREE version 2.0.3 [25] with the best-fitting evolutionary model and recording the best tree for each of the 1000 bootstrap alignment samples. The reference species tree was reconstructed from the concatenated alignment of 117 single-copy gene families, present as single-copy in all 31 species, with IQ-TREE version 2.0.3, with the best-fitting model for each partition in the concatenated alignment [25]. Conditional clade probabilities for each gene tree were calculated with ALEobserve [14], and the gene trees were reconciled against the species trees with ALEml_undated. Tree reconciliations were performed for each possible root branch in the unrooted species tree (59 in total), with different T:D ratios. In the first tree, reconciliations round gene transfer and gene duplication rates were freely estimated by ALE (1:1 T:D analysis). To achieve specific T:D ratios, additional tree reconciliation runs were carried out by setting gene transfer and gene duplication rates in the same manner as described for the bacterial dataset. The gene transfer rates for each gene tree were selected from the 1:1 T:D analysis and the gene duplication rates were adjusted accordingly as to obtain the desired T:D ratio. The T:D ratios tested for the fungi-metazoan dataset were 1:2, 1:50 and 50:1.

The approximately unbiased (AU) tests [16] were performed with custom R scripts using the ‘scaleboot’ library [26]. The Spearman correlation tests were performed in MATLAB. All tests were considered significant at *p*-value ≤ 0.05.

## 3. Results and Discussion

### 3.1. Gene Transfer to Gene Duplication Ratios Constrain the Number of Inferred Species Tree Roots

**Figure 1:**
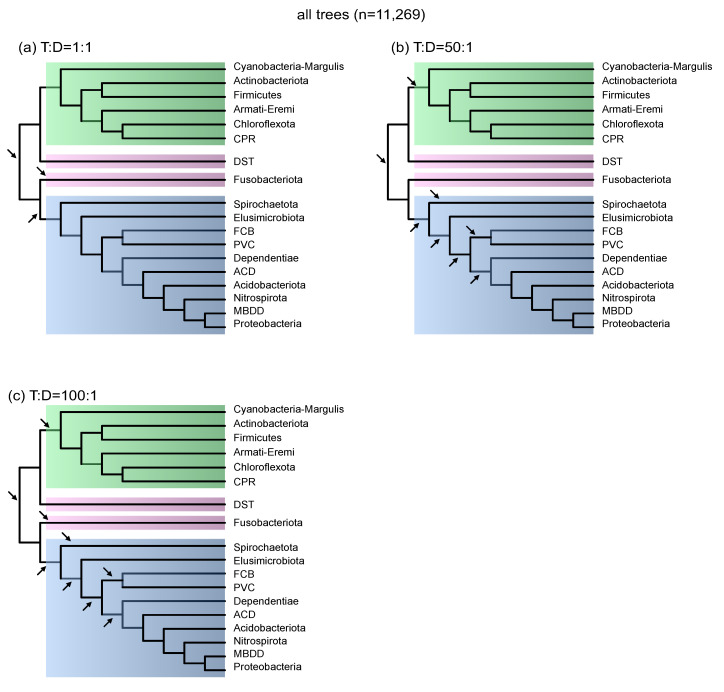
Rooting the bacterial species tree with ALE using different T:D settings. The species tree encompasses 265 bacterial species and was reconstructed from the concatenated alignment of 62 protein-coding genes using maximum-likelihood [17]. 62 alternative root positions within the tree were tested through the reconciliation of 11,269 maximum-likelihood gene trees and the most likely roots that passed the AU tests (*p* > 0.05) are indicated with black arrows. (**a**) analysis with 1:1 T:D ratio; (**b**) 50:1 T:D ratio; and (**c**) T:D ratio of 100:1. The clade in green shade corresponds to the Terrabacteria lineage, and the clade in blue shade corresponds to the Gracilicutes lineage.

To see what effect the use of a more realistic T:D ratio would have on the inference of the bacterial root, we set the T:D ratio to values that agree with previous studies [21,22], and repeated the analyses following the same protocol as in Coleman et al. [17]. As an initial control, we used exactly the same parameters as Coleman et al. [17] used, and allowed ALE to estimate T and D on its own (1:1 T:D ratio); this exactly reproduced the results of Coleman et al. [17] and found the same three bacterial roots that passed the AU tests (Figure 1). We then repeated the analyses, but changed T and D as to conform to a T:D ratio of 50:1 across gene trees, a ratio that is considered to be a conservative (lower-bound) estimate of T:D ratios for bacterial genes [22]. All that the 50:1 T:D setting does is to penalize bacterial gene duplications relative to bacterial gene transfers, because we used the same species tree (and remaining parameters) in comparison to the 1:1 T:D analysis. In contrast to the three roots obtained with the 1:1 T:D analysis, the 50:1 T:D ratio resulted in eight bacterial roots (Figure 1). Three roots fell between Gracilicutes and Terrabacteria, two of which were also obtained with the 1:1 T:D analysis. The remaining five roots identified with a T:D of 50:1 fell within the bacterial group that Coleman et al. [17] designate as Gracilicutes (Figure 1).

In an earlier study focusing on recent gene transfers and recent gene duplications, we found that average bacterial T:D ratios across genes can approach or exceed 100:1 in some bacterial lineages [22]. To understand what influence a higher T:D ratio would have on the root inferences, we repeated the tests by setting T and D across gene trees so as to conform to a 100:1 T:D ratio. The 100:1 analysis uncovered nine bacterial roots in total, that is, one more than the 50:1 T:D analysis. Four roots were found between Gracilicutes and Terrabacteria and five roots within Gracilicutes (Figure 1). Together, the 50:1 and 100:1 T:D analyses show that gene duplication and gene transfer rates have a significant effect upon ALE’s root choices.

In terms of trying to get realistic inferences, it is indeed possible that fixed T:D ratios across genes are too strict. That is because some genes, such as those involved in translation, are less frequently transferred [27,28]. Thus, we performed one additional analysis by capping the T:D ratio so as not to fall below 50:1, thereby allowing ALE to assume any T:D ratio that prefers gene transfers over gene duplications by at least a factor of 50:1. With this setting, the AU test recovered a total of ten bacterial roots: five falling between Gracilicutes and Terrabacteria; three roots that fall within Gracilicutes; and two roots that fall within the Terrabacteria, one of which separates a clade of Actinobacteria and Firmicutes from the rest of the tree (Supplemental Table S1). Our results reveal that more realistic T:D ratios increase the number of inferred roots for the bacterial tree. Permitting unconstrained variation of T:D across gene trees, as ALE does in the default 1:1 T:D setting, unnecessarily constrains the likelihoods assigned to alternative bacterial roots.

### 3.2. Imbalance of Gene Gains and Gene Losses

**Figure 2:**
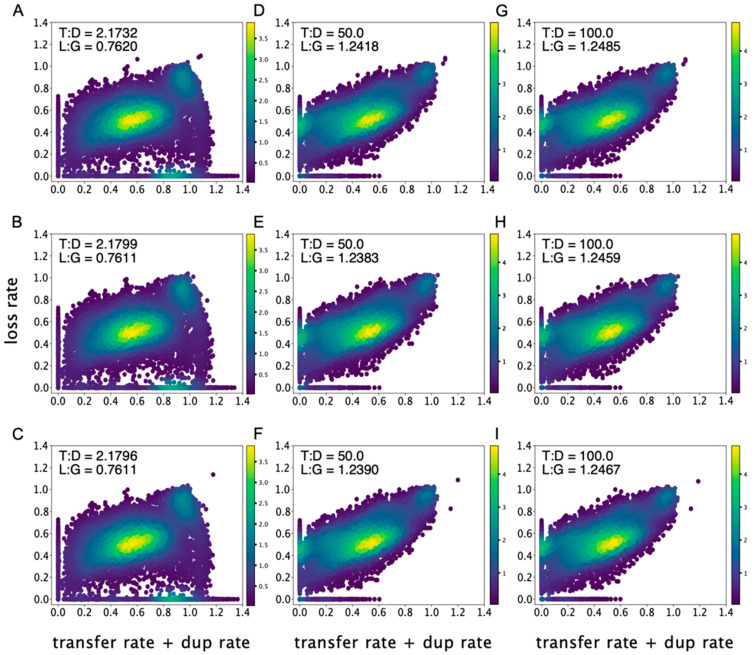
The rates of gene gains and gene losses across bacterial genes. The rates of gains (horizontal axis), measured as the sum of gene duplication and gene transfer rates, versus the rates of losses (vertical axis) as obtained with different T:D settings with ALE (columns) conditioned upon three alternative bacterial roots [17]. (**A**–**C**) 1:1 T:D ratio. (**D**–**F**) 50:1 T:D ratio. (**G**–**I**) 100:1 T:D ratio. Insets show the average transfer-to-duplication ratio (T:D) and loss-to-gain ratio (L:G) across 11,265 gene trees. The color bars indicate the number of gene trees (density).

Both gene duplications and gene transfers can contribute novel gene-copies, thereby steadily increasing genome size. However, bacterial genome sizes are not free to expand, they are themselves constrained [29] due to deletional bias [30,31] and energetic costs of gene expression [32]. Thus, in any realistic analysis with ALE, gene gains should be balanced out by gene losses in bacteria. Since in all our analyses we allowed ALE to automatically optimize gene loss rates, the distribution of gene loss rates across gene trees as a function of different T:D settings offers an independent means to assess the realism of ALE’s analyses. We compared the balance of gene gains and gene losses across bacterial genes, as obtained with different T:D settings (columns in Figure 2), conditioned on the three bacterial roots reported by Coleman et al. [17] (rows in Figure 2). The three panels of Figure 2 on the left (A–C) show the results for the 1:1 T:D analysis (default), the panels in the middle (D–F) show the results for the T:D of 50:1, and the panels at the right (G–I) show the results for the 100:1 T:D ratio. In all the different T:D settings the rates of gene loss for each gene tree were freely estimated by ALE and, as such, the distributions of gene loss rates offer an opportunity to evaluate the results. Gene gains and losses are more balanced, with narrower distribution, for the more realistic 50:1 and 100:1 T:D ratios in comparison to the 1:1 T:D ratio (Figure 2). The trend is largely independent upon the root position within the bacterial species tree (see also Supplemental Figure S1).

Dividing the mean loss rate (L) by the mean gain rate (G) across gene trees provided us with an L:G ratio across gene families, a proxy for the direction of genome size evolution. An L:G above one indicates genome reduction, whereas an L:G below one indicates genome growth. The body of literature showing that bacterial genomes lose genes more often than they acquire genes is rich [30,31,33,34,35,36,37]. In both the 50:1 and 100:1 T:D ratio analyses, gene losses do indeed exceed gene gains in bacteria, with L:G ratios of 1.24 and 1.25, respectively (Table 1). The L:G estimate is indicative of a general trend for an average bacterial genome relative to all genomes in the dataset. Exceptions to this obviously exist. For instance, filamentous cyanobacteria species exhibit larger genomes in comparison to their unicellular cyanobacteria relatives due to the increase in genome size at the origin of filaments [38,39,40,41]. ALE, in its default 1:1 T:D setting, indicates instead that the general tendency of an average bacterial genome is to become larger in size, yielding a low L:G value of 0.76 which, in other words, means that gene gains are 24% more frequent than gene losses, contrary to observations in real data. In other words, the 1:1 T:D analyses indicate that gene gains outnumber gene losses in bacterial genomes, in contradiction to previous observations [30,31,33,34,35,36,37].

### 3.3. Testing ALE on a Simple Phylogenetic Case: The Fungal-Metazoan Root

Demonstrating the limitations of species tree rooting with ALE using the bacterial tree carries the caveat that the bacterial root is not known nor is it known whether the species tree that Coleman et al. [17] used to search for roots is correct—an issue that we do not address here. To circumvent some of the unknowns underlying bacterial evolution, we tested the ability of ALE to recover the root of a simpler phylogenetic case: the root of the metazoan (animals) and fungal tree. By all accounts, fungi are monophyletic relative to metazoa and vice versa [4,42,43,44], with the root of the tree lying undisputedly on the branch splitting the two groups. We analyzed the ability of ALE to identify the fungal-metazoan root using a dataset composed of 31 species and 15,614 gene trees. We reconstructed a reference fungal-metazoan tree from the concatenated alignment of 117 single-copy genes shared among all 31 species using maximum-likelihood and tested each of the 59 branches in the unrooted tree as a possible root position, using different T:D ratios with ALE.

**Figure 3:**
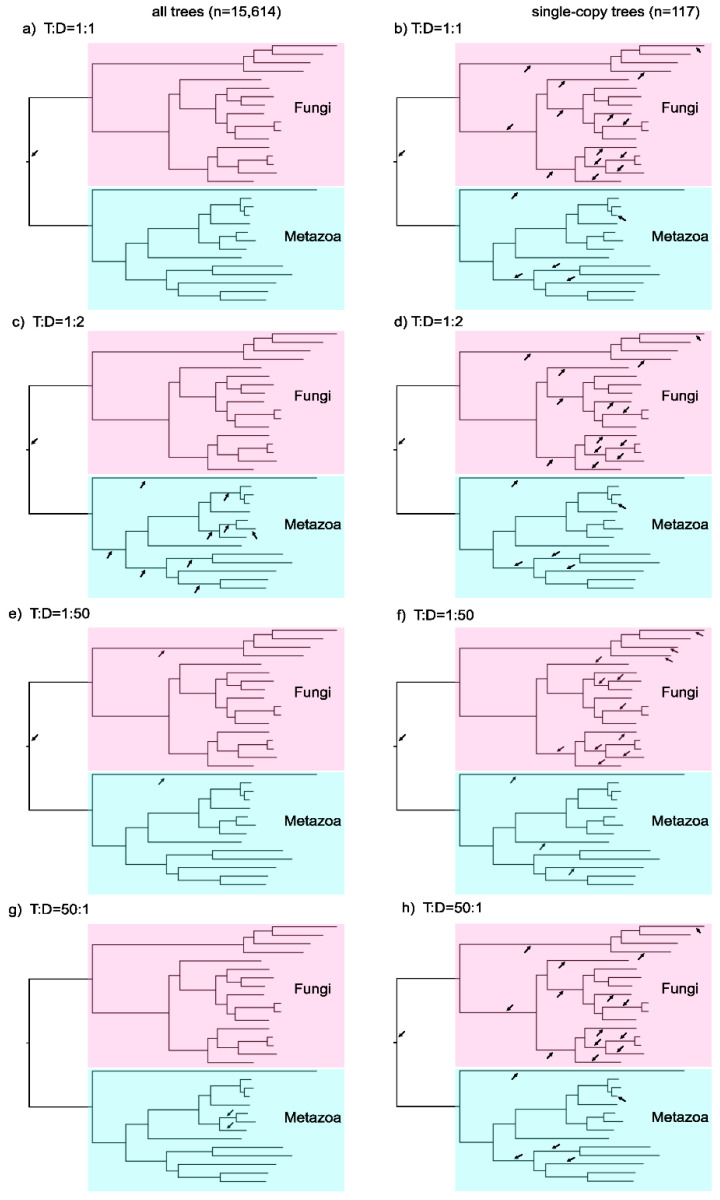
Inferences of the fungi-metazoan species tree with ALE. The tree was reconstructed via maximum-likelihood analyses of 117 concatenated protein-coding genes spanning 31 species (for complete species composition see Supplemental Table S3). The species tree is shown rooted on the known root branch that separates fungi (**pink**) from metazoan (**light green**). The rooting analyses were performed under different T:D settings (rows). The reconciliations were performed for 15,614 maximum-likelihood gene trees against the all-possible rooted versions of the unrooted tree (59 roots in total). The results for the AU test are shown separately for all gene trees [**left**; (**a**,**c**,**e**,**g**)], and for 117 gene trees that contain all species without paralogs [**right**; (**b**,**d**,**f**,**h**)], referred in the text as single-copy gene trees. The root branches that passed the AU test (*p* > 0.05) are marked with black arrows.

Assuming an initial T:D ratio of 1:1, the AU tests identified the correct root (Figure 3). By inspecting the evolutionary rates across gene trees, we found that the average number of duplications (D) was twice as high as the average number of transfers (T) across genes, that is an average T:D ratio of 1:2 (Table 2). This result contrasts with a previous estimate of a 1:100 T:D ratio in eukaryotes [44] based on a large sample of eukaryotic gene families harboring gene duplications. It was observed that fewer than 1% of all eukaryotic gene duplications are shared among eukaryotic supergroups [44]. On the other hand, 99% of the gene duplications are exclusively found in a single eukaryotic supergroup, most of them exclusive to plants, metazoa or fungi [44]. These observations suggest an approximate T:D ratio of 1:100 in eukaryotes, assuming that gene transfers are the *sole* evolutionary process responsible for generating duplicates shared among eukaryotic supergroups (see [44] for further discussion). However, assuming gene transfer as the sole process is certainly an extreme proposition, and thus the T:D value of 1:100 only serves as a very liberal reference for the analyses of the fungi-metazoan dataset. Interestingly, for the 117 single-copy genes used to infer the fungi-metazoan tree, ALE calculated an abnormally high average T:D ratio of 26,968:1. According to ALE, there are only two possible causes for these tree incongruences: gene transfers or gene duplications. Other explanations are theoretically possible, such as incomplete lineage sorting and tree reconstruction errors, but those factors are not part of the evolutionary model that ALE implements. Topological discordances among gene trees and species trees are expected to occur in real biological data as a result of the ever-present problem of tree reconstruction errors. However, ALE assumes that both gene tree and species tree are correct, a rather unrealistic assumption which is known to introduce biases in tree reconciliation analyses [45]. Although phylogenetic errors may be accounted for by collapsing branches in the trees with bootstrap values below a pre-defined threshold, this is neither a common practice nor guaranteed to alleviate biases arising from tree reconstruction errors, since even branches with high bootstraps may be incorrect. According to the model implemented in ALE, there are two possibilities to explain topological deviations in the fungal-metazoan single-copy gene trees: ‘gene duplication’ or ‘gene transfers’. Calling gene duplications would imply a series of parallel gene losses which penalize the likelihood score of the ‘gene duplication’ scenario to a suboptimal value in comparison to the ‘gene transfer’ scenario, inflating gene transfer rates to an unrealistic degree.

Distinguishing between gene transfers and gene duplications is only possible if T and D are known. Whether the values of T and D estimated by ALE are accurate or not is a crucial aspect of tree reconciliation analyses that we address here. Our results suggest that the T:D ratios estimated by ALE, when left to its own devices, are abnormal in a dataset-independent manner. The frequency of gene duplications and gene transfers, and their ratios, are known to be very different in bacteria and in eukaryotes. Yet ALE estimates that the average T:D ratio for the fungal-metazoan genes is roughly 1:2 and 2:1 for bacterial genes, both average T:D very close to the initial 1:1 T:D ratio used as prior. The ranges of T:D ratios across genes are also too wide, ranging from 10^−11^ to 10^9^ across fungal-metazoan genes and 10^−11^ to 10^10^ across bacterial genes (Supplemental Figure S1).

The single-copy gene trees from the fungi-metazoan dataset offer an opportunity to further demonstrate how the T and D rates affect the root inferences. Performing the AU tests using single-copy trees alone under 1:1 T:D leads to an inference of 20 equally supported roots for the fungal-metazoan tree (Figure 3). Why can the single-copy gene trees not retrieve the correct root, while all gene trees combined can? One possibility is that the sample size of 117 gene trees is not large enough. We ruled out sample size as the main issue because the correct root was recovered with a random sample of 117 gene trees (Supplemental Table S2). The most plausible explanation has to do with the relative importance of gene duplications and gene transfers for species tree rooting, as we explain in the following.

### 3.4. Gene Duplications Are More Informative Than Gene Transfers for Rooting Species Trees

ALE’s ability to root species trees rests upon gene duplications, gene transfers and gene losses, but the relative contribution of each of these types of events for ALE’s rooting procedure is obscure. It has been speculated that gene transfers are the main phylogenetic information that enables the rooting with ALE because gene transfers constrain the relative age of donor-recipient lineage pairs [17]. Since gene transfers cannot happen forward or backward in time, gene transfer would hold the potential to polarize node pairs in the species tree that correspond to donor-recipient lineages. While this argument may hold for dated species trees, that is, trees for which the branch lengths are proportional to time, gene transfers have only a limited potential to root undated species trees, which are most commonly used in phylogenetic practice.

**Figure 4:**
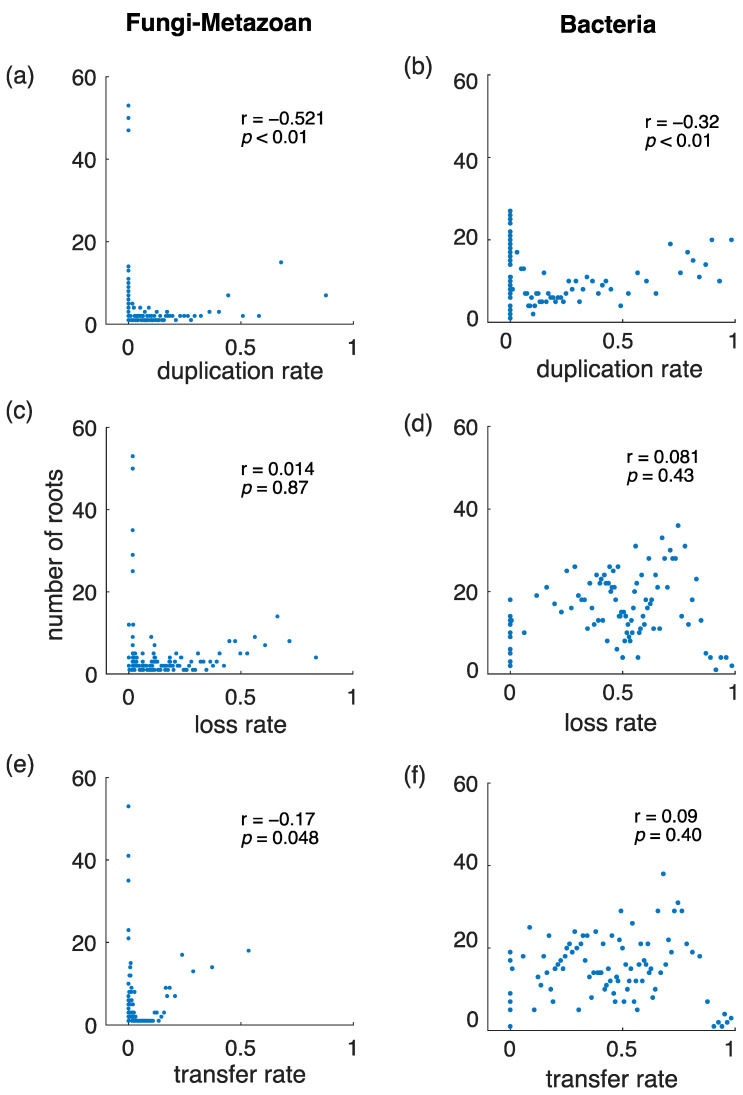
Power analyses for the number of inferred roots as a function of varying evolutionary rates. Gene trees were ranked according to the evolutionary rates for gene duplication (D) (**a**,**b**), gene transfer (T) (**c**,**d**) and gene loss (L) (**e**,**f**) independently (estimated autonomously by ALE, referred in the text as 1:1 T:D ratio). The AU tests were performed interactively for all non-overlapping sets of consecutive 100 gene trees in the ranked list. The number of significant roots (vertical axis) were plotted against the mean evolutionary rates of the gene trees (horizontal axis). The insets show the correlation coefficient (r) and *p*-value (*p*) from the two-tailed Spearman correlation tests. Gene duplication rates, not gene transfer rates, have the strongest negative correlation with the number of inferred roots.

To investigate the issue, we performed the following experiment for gene duplication, gene transfer and gene loss independently: We ranked gene trees in decreasing order of evolutionary rates for the given variable (gene duplication rate, gene transfer rate, and gene loss rate), as freely estimated by ALE (1:1 T:D), and selected the top 100 gene trees in each ranked list to carry out the AU tests for rooting. The number of significant roots obtained were counted and the AU tests were iteratively repeated for the following 100 gene trees in the ranked list until all non-overlapping subsets 100 gene trees were analyzed. The number of significant roots obtained as a function of the gene duplication rate alone, for both the bacterial and fungal-metazoan datasets, are shown in Figure 4. This simple power analysis clearly shows that gene duplications, not gene transfers, are the most informative type of phylogenetic event for rooting species trees with ALE, a trend observed for both the fungal-metazoan tree and the bacterial tree. The number of significant roots is negatively correlated with the average gene duplication rate across the gene trees (rho = −0.32 and *p* < 0.01 for bacteria; rho = −0.52 and *p* < 0.01 for fungi-metazoan; two-tailed Spearman correlation). On the other hand, the average gene loss rate has no significant influence on the number of inferred roots (rho ≈ 0.08 and *p* ≈ 0.4 for bacteria; rho ≈ 0.01 and *p* ≈ 0.9 for fungi-metazoan), while the gene transfer rate shows a weak correlation with the number of roots which is only marginally significant (rho = 0.09 and *p* = 0.04 for bacteria; rho = −0.17 *p* ≈ 0.05, for fungi-metazoan). Note that the Spearman correlation is a non-parametric statistic, as opposed to the Pearson correlation, and is appropriate for identifying dependencies between variables even when the underlying distribution is unknown.

The fact that gene duplications are more important than gene transfer for the species tree rooting with ALE readily explains why more realistic T:D ratios increase the number of inferred roots for the bacterial phylogeny. Higher T:D ratios result in fewer gene duplications, rendering the root inference more uncertain. The T:D ratio serves the purpose of determining the proportion of tree incongruences that are attributed to gene duplications and the proportion of tree incongruences that are attributed to gene transfers. For the bacterial dataset, the optimized T:D ratios were on average 2:1, which means that approximately 33% of the tree incongruences in the data were attributed to gene duplications, whereas the remaining 67% were attributed to gene transfers. However, most of these gene duplications are in fact false gene duplications, as evidenced by the unexpectedly large average T:D of 2:1. Due to the high number of false gene duplications, incorrect roots in the bacterial tree receive inappropriately high statistical support. In other words, the evolutionary model is biased and not as powerful as it may seem at first sight (due to the high statistical support). By increasing T:D, the number of false gene duplications is reduced. As a consequence, the evolutionary model becomes less biased and the root inference appears more uncertain (as it should), because the paucity of true gene duplications in the data renders the identification of the correct root not possible. In tree the reconciliation analyses performed by ALE, the finite number of tree incongruences between gene trees and the species tree constrains *a priori* the total number of evolutionary events (gene transfers plus gene duplications) that can be invoked. Importantly, the number of incongruences between a gene tree and the species tree depends upon the root position in the species tree such that species tree roots that induce a larger number of tree incongruences will generally attain smaller likelihood scores in comparison to roots that induce fewer tree incongruences.

The importance of gene duplications for rooting furthermore explains why the single-copy gene trees alone cannot retrieve the correct root for the fungal-metazoan tree. Single-copy genes bear few detectable gene duplications and as such are not root informative. The importance of gene duplications on the root choice by ALE becomes even clearer when we carry out the analyses fixing the T:D ratio in such a way that we allow for more gene duplications to occur in comparison to gene transfers. Using the fungal-metazoan tree as a clear reference, and increasing the T:D ratio to 1:2, as opposed to the default 1:1, the AU tests identified 19 roots when using only the single-copy gene trees to root the fungal-metazoan tree, one root less than is obtained with the 1:1 T:D analyses in which the occurrences of gene duplications are more restricted. Allowing even more gene duplications by setting ALE to a T:D of 1:50, the number of roots decreases to 16 (Figure 3). Overall, these analyses indicate that allowing more gene duplications in ALE increases the power of the AU test in rejecting incorrect roots. Yet gene duplications are so rare across the single-copy genes that a decisive root inference for the fungal-metazoan tree is not possible with the single-copy gene trees alone. Furthermore, our observation that the analysis of all fungi-metazoan gene trees with T:D set to 1:1 recovers the true root makes sense because gene duplications are abundant in eukaryotic genomes, in particular in fungi and metazoa [38]. ALE is able to find a sufficient number of the gene duplications present across all fungi-metazoan gene trees, yet ALE infers a high number of false gene transfers because the average T:D ratio of 1:2 is too high even in comparison to the very liberal reference T:D value of 1:100 [38]. Despite the high number of falsely inferred gene transfers in the fungi-metazoan data, there is no considerable bias for the root inference because gene transfers are not root informative, as we have shown. Interestingly, the analyses of all fungi-metazoan gene trees with a T:D set of 1:2 and 1:50, in which more gene duplications can be accommodated in comparison to the default 1:1, renders an uncertain root inference for the fungi-metazoan phylogeny. The reason is likely due to a systematic tendency of tree reconciliations to incorrectly place terminal gene duplications at the base of gene trees [45], a problem that becomes more acute when more gene duplications are allowed (other factors, however, may also play a role).

If gene duplications indeed play a crucial role for the root inferences, then penalizing the occurrence of gene duplications and facilitating the occurrence of gene transfers instead should have a negative effect on the inferences. To investigate this possibility, we set the T:D ratio for the fungal-metazoan gene trees to an unrealistic 50:1 T:D, whereby gene transfers exceed gene duplications by a factor of 50. With a T:D ratio of 50:1, we obtained two equally supported roots for the fungal-metazoan tree, both of which lie within the metazoan lineage, far from the true root branch (Figure 3).

## 4. Conclusions

We have demonstrated that the rooting of species trees with ALE depends on the quality of the gene transfer to gene duplication ratio used as input. The reliance on hard-coded priors of 1:1 used to estimate the relative evolutionary rates of duplications and transfers hinders root inference because evolutionary rates vary considerably across lineages. The influence of prior choice as source of bias in phylogenetic inferences has been reported before [46].

From the theoretical point of view, tree reconciliation methods are an attempt to fully model the complex evolutionary process shaping the evolution of genes and genomes. In a single gene-tree-species-tree reconciliation analysis, there are several parameters that need to be simultaneously estimated besides T:D—effective population size, fraction of non-sampled lineages, gene loss rate, and gene tree root (which is also optimized in ALE). Each of these parameters are required for the analyses and are likely to affect the accuracy of species tree rooting. Investigating all of these parameters is out of our scope. We purposefully focused on T:D ratios in particular because we have a good reference to the distribution of actual T:D values. A realistic distribution of bacterial T:D values were obtained by an independent study [22] which explicitly minimized biases by focusing on inferences of T:D using recent evolutionary events, for which the distinction among gene transfers and gene duplications is possible without committing to any particular bacterial species tree (hence a reconciliation-free approach). While we specifically focused on the effect of T:D here, our work will motivate further investigations about the accuracy of species tree rooting obtained with tree reconciliation methods.

Our work goes well beyond previous studies that assessed the performance of tree reconciliation models with simulations. Simulations, despite being useful, present limitations that are hard to bypass: (i) they do not fully capture the characteristics of biological data and are highly dependent on parameter choice; (ii) tree reconstruction errors are often not accounted for; and (iii) the evolutionary models used to carry out simulations often belong to the same family of the models being tested, making the study biased by design.

We observed that in using realistic, independently estimated T:D ratios to infer the bacterial root with ALE, the inferred root placements become more uncertain and the number of possible roots increases relative to the results of ALE left to its own devices. It is notable that gene duplications are significantly more important than gene transfers for rooting. Gene duplications are vertically inherited and, as such, are rich phylogenetic characters to trace the evolution of species [44,47], whereas frequent gene transfers, such as those observed among prokaryotes, are not root informative. Indeed, it has been suggested that the high frequency of gene transfers among prokaryotes may render the species tree framework inapplicable [48,49], a proposition that is nevertheless challenging to implement in practice. Overall, our comprehensive analysis for the bacterial tree does not strongly support the Gracilicutes-Terrabacteria root. Furthermore, we show that root inferences obtained through gene-tree-species-tree reconciliations are far more uncertain than previously recognized due to factors that are only now coming to light.

## Figures and Tables

**Table 1:** Summary statistics across all 11,272 bacterial gene trees for evolutionary rates obtained with different T:D ratios. Gains were defined as the sum of transfers and duplication rates.

	Transfer Rate			Duplication Rate			Loss Rate			Gain Rate			Loss/Gain Ratio
Dataset	Mean	Median	Std	Mean	Median	Std	Mean	Median	Std	Mean	Median	Std	
1:1	4.18 × 10^−1^	4.30 × 10^−1^	2.72 × 10^−1^	1.93 × 10^−1^	5.53 × 10^−2^	2.82 × 10^−1^	4.65 × 10^−1^	4.96 × 10^−1^	2.59 × 10^−1^	6.12 × 10^−1^	6.14 × 10^−1^	2.85 × 10^−1^	7.59 × 10^−1^
50:1	4.18 × 10^−1^	4.30 × 10^−1^	2.72 × 10^−1^	8.4 × 10^−3^	8.6 × 10^−3^	5.4 × 10^−3^	5.29 × 10^−1^	5.25 × 10^−1^	2.08 × 10^−1^	4.27 × 10^−1^	4.39 × 10^−1^	2.77 × 10^−1^	1.23 × 10^0^
100:1	4.18 × 10^−1^	4.30 × 10^−1^	2.72 × 10^−1^	4.2 × 10^−3^	4.3 × 10^−3^	2.7 × 10^−3^	5.27 × 10^−1^	5.25 × 10^−1^	2.07 × 10^−1^	4.23 × 10^−1^	4.35 × 10^−1^	2.75 × 10^−1^	1.24 × 10^0^
50:1 or more	9.91 × 10^0^	2.75 × 10^0^	1.39 × 10^1^	1.93 × 10^−1^	5.53 × 10^−2^	2.82 × 10^−1^	5.51 × 10^0^	1.15 × 10^0^	8.02 × 10^0^	1.01 × 10^1^	2.81 × 10^0^	1.42 × 10^1^	5.45 × 10^−1^

**Table 2:** Summary statistics across 15,614 fungi-metazoan gene trees obtained with different T:D ratios. Gains were defined as the sum of transfers and duplication rates. The statistics for 117 universal single-copy (sc) gene trees are shown separately at the bottom rows.

	Transfer Rate			Duplication Rate			Loss Rate			Gain Rate			Loss/Gain Ratio
Dataset	Mean	Median	Std	Mean	Median	Std	Mean	Median	Std	Mean	Median	Std	
1:1	3.56 × 10^−2^	1.00 × 10^−6^	7.73 × 10^−2^	7.30 × 10^−2^	5.67 × 10^−6^	1.29 × 10^−1^	1.40 × 10^−1^	8.81 × 10^−2^	1.67 × 10^−1^	1.08 × 10^−1^	5.54 × 10^−2^	1.62 × 10^−1^	1.29 × 10^1^
1:2	3.56 × 10^−2^	1.00 × 10^−6^	7.73 × 10^−2^	7.21 × 10^−2^	2.00 × 10^−6^	1.54 × 10^−1^	1.54 × 10^−1^	8.59 × 10^−2^	2.22 × 10^−1^	1.06 × 10^−1^	3.00 × 10^−6^	2.31 × 10^−1^	1.44 × 10^1^
1:50	3.56 × 10^−2^	1.00 × 10^−6^	7.73 × 10^−2^	1.77 × 10^1^	5.00 × 10^−5^	3.86 × 10^1^	1.15 × 10^1^	1.71 × 10^−1^	2.42 × 10^1^	1.81 × 10^1^	5.1 × 10^−5^	3.94 × 10^1^	6.36 × 10^−1^
50:1	3.56 × 10^−2^	1.00 × 10^−6^	7.73 × 10^−2^	7.0 × 10^−4^	2.00 × 10^−8^	1.5 × 10^−3^	1.27 × 10^−1^	8.52 × 10^−2^	1.42 × 10^−1^	3.63 × 10^−2^	1.02 × 10^−6^	7.89 × 10^−2^	3.52 × 10^1^
100:1	3.56 × 10^−2^	1.00 × 10^−6^	7.73 × 10^−2^	4.0 × 10^−4^	1.00 × 10^−8^	8.0 × 10^−4^	1.27 × 10^−1^	8.44 × 10^−2^	1.41 × 10^−1^	3.59 × 10^−2^	1.00 × 10^−6^	7.81 × 10^−2^	3.54 × 10^1^
1:1 (sc)	4.8 × 10^−3^	1.00 × 10^−10^	9.3 × 10^−3^	2.26 × 10^−7^	1.0 × 10^−10^	6.34 × 10^−7^	4.6 × 10^−3^	1.0 × 10^−10^	9.4 × 10^−3^	4.8 × 10^−3^	2.0 × 10^−10^	9.3 × 10^−3^	9.58 × 10^−1^
1:2 (sc)	4.8 × 10^−3^	1.00 × 10^−10^	9.3 × 10^−3^	9.7 × 10^−3^	2.0 × 10^−10^	1.87 × 10^−2^	5.4 × 10^−3^	1.0 × 10^−10^	1.18 × 10^−2^	1.45 × 10^−2^	3.0 × 10^−10^	2.80 × 10^−2^	3.72 × 10^−1^
1:50 (sc)	4.8 × 10^−3^	1.00 × 10^−10^	9.3 × 10^−3^	2.42 × 10^−1^	5.00 × 10^−9^	4.66 × 10^−1^	9.69 × 10^−2^	1.0 × 10^−10^	2.27 × 10^−1^	2.47 × 10^−1^	5.1 × 10^−9^	4.75 × 10^−1^	3.92 × 10^−1^
50:1 (sc)	4.8 × 10^−3^	1.00 × 10^−10^	9.3 × 10^−3^	9.69 × 10^−5^	2.0 × 10^−12^	2.0 × 10^−4^	4.6 × 10^−3^	1.0 × 10^−10^	9.4 × 10^−3^	4.9 × 10^−3^	1.0 × 10^−10^	9.5 × 10^−3^	9.38 × 10^−1^
100:1 (sc)	4.8 × 10^−3^	1.00 × 10^−10^	9.3 × 10^−3^	4.84 × 10^−5^	1.0 × 10^−12^	9.33 × 10^−5^	4.6 × 10^−3^	1.0 × 10^−10^	9.4 × 10^−3^	4.9 × 10^−3^	1.0 × 10^−10^	9.4 × 10^−3^	9.38 × 10^−1^

## Data Availability

All tree reconciliation files generated in this study are fully available under: https://doi.org/10.6084/m9.figshare.19697500.v1 (accessed on 30 June 2022).

## Supplementary Materials

The following supporting information can be downloaded at: https://www.mdpi.com/article/10.3390/life12070995/s1. Figure S1: The rates of gene gains and gene losses across bacterial genes for bacterial roots (rows) that were found with a T:D ratio of 50:1 and 100:1 and not reported earlier by Coleman et al. [17]. The columns represent the different T:D settings. Table S1: AU tests for all bacterial roots with different T:D settings. Table S2: AU tests for all fungal-metazoan roots with different T:D settings. Table S3: Complete species composition of the fungal-metazoan analysis.

## References

[1] Kluge A.G., Farris J.S. (1969). Quantitative phyletics and the evolution of anurans. Syst. Zool..

[2] Brown J.R., Doolittle W.F. (1995). Root of the universal tree of life based on ancient aminoacyl-tRNA synthetase gene duplications. Proc. Natl. Acad. Sci. USA.

[3] Farris J.S. (1972). Estimating phylogenetic trees from distance matrices. Am. Nat..

[4] Tria F.D.K., Landan G., Dagan T. (2017). Phylogenetic rooting using minimal ancestor deviation. Nat. Ecol. Evol..

[5] Lepage T., Bryant D., Philippe H., Lartillot N. (2007). A general comparison of relaxed molecular clock models. Mol. Biol. Evol..

[6] Williams T.A., Heaps S.E., Cherlin S., Nye T.M.W., Boys R.J., Embley T.M. (2015). New substitution models for rooting phylogenetic trees. Philos. Trans. R. Soc. B Biol. Sci..

[7] Graham S.W., Olmstead R.G., Barrett S.C.H. (2002). Rooting phylogenetic trees with distant outgroups: A case study from the commelinoid monocots. Mol. Biol. Evol..

[8] Huelsenbeck J.P., Bollback J.P., Levine A.M. (2002). Inferring the root of a phylogenetic tree. Syst. Biol..

[9] Wade T., Thiberio Rangel L., Kundu S., Fournier G.P., Bansal M.S. (2020). Assessing the accuracy of phylogenetic rooting methods on prokaryotic gene families. PLoS ONE.

[10] Lamarca A.P., Mello B., Schrago C.G. (2022). The performance of outgroup-free rooting under evolutionary radiations. Mol. Phylogenet. Evol..

[11] Goodman M., Czelusniak J., Moore G.W., Romero-Herrera A.E., Matsuda G. (1979). Fitting the gene lineage into its species lineage, a parsimony strategy illustrated by cladograms constructed from globin sequences. Syst. Zool..

[12] Page R.D.M. (1994). Maps between trees and cladistic analysis of historical associations among genes, organisms, and areas. Syst. Biol..

[13] Bansal M.S., Alm E.J., Kellis M. (2012). Efficient algorithms for the reconciliation problem with gene duplication, horizontal transfer and loss. Bioinformatics.

[14] Szöllosi G.J., Rosikiewicz W., Boussau B., Tannier E., Daubin V. (2013). Efficient exploration of the space of reconciled gene trees. Syst. Biol..

[15] Doyon J., Ranwez V., Daubin V., Berry V. (2011). Models, algorithms and programs for phylogeny reconciliation. Brief. Bioinform..

[16] Shimodaira H. (2002). An approximately unbiased test of phylogenetic tree selection. Syst. Biol..

[17] Coleman G.A., Davín A.A., Mahendrarajah T.A., Szánthó L.L., Spang A., Hugenholtz P., Szöllősi G.J., Williams T.A. (2021). A rooted phylogeny resolves early bacterial evolution. Science.

[18] Xavier J.C., Gerhards R.E., Wimmer J.L.E., Brueckner J., Tria F.D.K., Martin W.F. (2021). The metabolic network of the last bacterial common ancestor. Commun. Biol..

[19] Pál C., Papp B., Lercher M.J. (2005). Adaptive evolution of bacterial metabolic networks by horizontal gene transfer. Nat. Genet..

[20] Dagan T., Artzy-Randrup Y., Martin W.F. (2008). Modular networks and cumulative impact of lateral transfer in prokaryote genome evolution. Proc. Natl. Acad. Sci. USA.

[21] Treangen T.J., Rocha E.P.C. (2011). Horizontal transfer, not duplication, drives the expansion of protein families in prokaryotes. PLoS Genet..

[22] Tria F.D.K., Martin W.F. (2021). Gene duplications are at least 50 times less frequent than gene transfers in prokaryotic genomes. Genome Biol. Evol..

[23] Huerta-Cepas J., Szklarczyk D., Forslund K., Cook H., Heller D., Walter M.C., Rattei T., Mende D.R., Sunagawa S., Kuhn M. (2016). EGGNOG 4.5: A hierarchical orthology framework with improved functional annotations for eukaryotic, prokaryotic and viral sequences. Nucleic Acids Res..

[24] Katoh K., Standley D.M. (2013). MAFFT multiple sequence alignment software version 7: Improvements in performance and usability. Mol. Biol. Evol..

[25] Nguyen L.T., Schmidt H.A., von Haeseler A., Minh B.Q. (2015). IQ-TREE: A fast and effective stochastic algorithm for estimating maximum-likelihood phylogenies. Mol. Biol. Evol..

[26] Shimodaira H. (2008). Testing regions with nonsmooth boundaries via multiscale bootstrap. J. Stat. Plan Inference.

[27] Nagies F.S.P., Brueckner J., Tria F.D.K., Martin W.F. (2020). A spectrum of verticality across genes. PLoS Genet..

[28] Cohen O., Pupko T. (2010). Inference and characterization of horizontally transferred gene families using stochastic mapping. Mol. Biol. Evol..

[29] Dagan T., Martin W.F. (2007). Ancestral genome sizes specify the minimum rate of lateral gene transfer during prokaryote evolution. Proc. Natl. Acad. Sci. USA.

[30] Ochman H., Lawrence J.G., Groisman E.A. (2000). Lateral gene transfer and the nature of bacterial innovation. Nature.

[31] Sela I., Wolf Y.I., Koonin E.V. (2016). Theory of prokaryotic genome evolution. Proc. Natl. Acad. Sci. USA.

[32] Lane N., Martin W.F. (2010). The energetics of genome complexity. Nature.

[33] Koonin E.V., Makarova K.S., Aravind L. (2001). Horizontal Gene Transfer in Prokaryotes: Quantification and Classification. Dict. Genom. Transcr. Proteom..

[34] Koonin E.V., Wolf Y.I. (2008). Genomics of bacteria and archaea: The emerging dynamic view of the prokaryotic world. Nucleic Acids Res..

[35] Snel B., Bork P., Huynen M.A. (2002). Genomes in flux: The evolution of Archaeal and Proteobacterial gene content. Genome Res..

[36] Mirkin B.G., Fenner T.I., Galperin M.Y., Koonin E.V. (2003). Algorithms for computing parsimonious evolutionary scenarios for genome evolution, the last universal common ancestor and dominance of horizontal gene transfer in the evolution of prokaryotes. BMC Evol. Biol..

[37] Puigbò P., Lobkovsky A.E., Kristensen D.M., Wolf Y.I., Koonin E.V. (2014). Genomes in turmoil: Quantification of genome dynamics in prokaryote supergenomes. BMC Med..

[38] Larsson J., Nylander J.A.A., Bergman B. (2011). Genome fluctuations in cyanobacteria reflect evolutionary, developmental and adaptive traits. BMC Evol. Biol..

[39] Stucken K., John U., Cembella A., Murillo A.A., Soto-Liebe K., Fuentes-Valdés J.J., Friedel M., Plominsky A.M., Vásquez M., Glöckner G. (2010). The smallest known genomes of multicellular and toxic cyanobacteria: Comparison, minimal gene sets for linked traits and the evolutionary implications. PLoS ONE.

[40] Herdman M., Janvier M., Rippka R., Stanier R.Y. (1979). Genome size of cyanobacteria. J. Gen. Microbiol..

[41] Hammerschmidt K., Landan G., Tria F.D.K., Alcorta J., Dagan T. (2021). The order of trait emergence in the evolution of cyanobacterial multicellularity. Genome Biol. Evol..

[42] Stechmann A., Cavalier-Smith T. (2002). Rooting the eukaryote tree by using a derived gene fusion. Science.

[43] Katz L.A., Grant J.R., Parfrey L.W., Burleigh J.G. (2012). Turning the crown upside down: Gene tree parsimony roots the eukaryotic tree of life. Syst. Biol..

[44] Tria F.D.K., Brueckner J., Skejo J., Xavier J.C., Kapust N., Knopp M., Wimmer J.L.W., Nagies F.S.P., Zimorski V., Gould S.B. (2021). Gene duplications trace mitochondria to the onset of eukaryote complexity. Genome Biol. Evol..

[45] Hahn M.W. (2007). Bias in phylogenetic tree reconciliation methods: Implications for vertebrate genome evolution. Genome Biol..

[46] Rannala B. (2002). Identifiability of parameters in MCMC Bayesian inference of phylogeny. Syst. Biol..

[47] Emms D.M., Kelly S. (2017). STRIDE: Species tree root inference from gene duplication events. Mol. Biol. Evol..

[48] Doolittle W.F., Bapteste E. (2007). Pattern pluralism and the Tree of Life hypothesis. Proc. Natl. Acad. Sci. USA.

[49] Bapteste E., O’Malley M.A., Beiko R.G., Ereshefsky M., Gogarten J.P., Franklin-Hall L., Lapointe F., Dupré J., Dagan T., Boucher Y. (2009). Prokaryotic evolution and the tree of life are two different things. Biol. Direct..

