# Supplementary figures and images for: Realistic Gene Transfer to Gene Duplication Ratios Identify Different Roots in the Bacterial Phylogeny Using a Tree Reconciliation Method
Source: Life (Basel). 2022 Jul 4;12(7):995. doi: 10.3390/life12070995 (PMC9322720; doi:10.3390/life12070995)

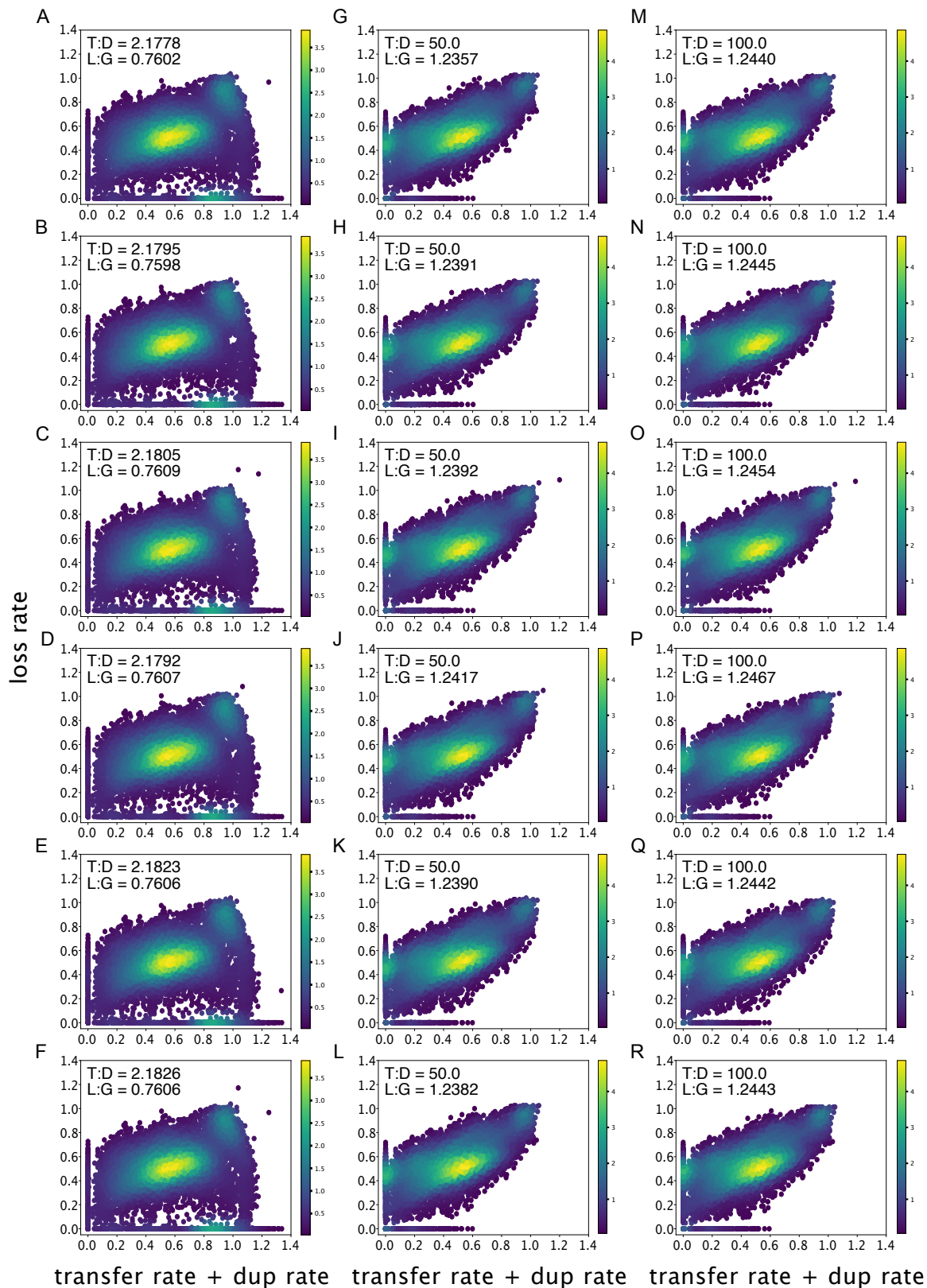

Supplement: Supplementary file 1 [file life-12-00995-s001.zip › Figure S1.pdf]
